# Psychiatric symptoms and COVID-19, the importance of differential diagnosis. about two cases

**DOI:** 10.1192/j.eurpsy.2021.724

**Published:** 2021-08-13

**Authors:** M. ValverDe Barea, M.O. Solis, L. Soldado Rodriguez, A. España Osuna

**Affiliations:** 1 Jaén, Complejo Hospitalario Jaén, Jaén, Spain; 2 Mental Health Unit, Complejo Hospitalario de Jaen, Jaen, Spain

**Keywords:** Psychiatric symptoms, behavioral disturbances, confusional syndrome, COVID-19

## Abstract

**Introduction:**

The COVID-19 pandemic presents symptomatic heterogeneity, so the differential diagnosis is even more relevant and more in patients with mental disorders. COVID-19 is a new disease that is under study and affects people over 65 with the greatest severity worldwide. The most frequent psychiatric symptoms are behavioral disturbances and confusional syndrome among those affected.

**Objectives:**

The objective is to demonstrate the importance of differential diagnosis in patients with psychiatric symptoms and covid-19.

**Methods:**

Patients aged 71 and 77, admitted to psychiatry. They present drowsiness that alternates with episodes of psychomotor agitation in which they verbalize fear of the coronavirus. Personal history: bipolar disorder and schizoaffective disorder. Psychopathological exploration: Spatial-temporal disorientation, uncooperative, fluctuating state of consciousness, verborrheic, salty and incoherent speech at times. Dysphoric mood. Psychomotor restlessness predominantly at night, verbal heteroaggressiveness. Negative to ingestion due to odynophagia. Sensory-perceptual alterations and nihilistic delusions “the virus has killed me, I’m already dead.” Upon admission, they present a cough and fever and are treated with azithromycin and dexamethasone for suspected COVID-19. Complementary tests: chest X-ray bilateral pleural effusion. Cranial CT: Diffuse cortical and subcortical brain parenchyma retraction pattern. PCR positive coronavirus.

**Results:**

After overcoming the infection and with psychopharmacological treatment the confusional syndrome remitted.

**Conclusions:**

Confusional syndrome can present with different psychiatric symptoms, so the differential diagnosis is very important and even more so in patients older than 65 years who present somatic pathologies or acute infections. The differential diagnosis of confusional syndrome is key to adequate treatment and favor the prognosis.

